# Pregnancy, child bearing and prevention of giving birth to the affected children in patients with primary immunodeficiency disease; a case-series

**DOI:** 10.1186/s12884-018-1927-6

**Published:** 2018-07-11

**Authors:** Saba Sheikhbahaei, Roya Sherkat, Nadezhda Camacho-Ordonez, Razie Khoshnevisan, Asadollah Kalantari, Mansour Salehi, Seyed Saman Nazemian, Mohammad Hossein Nasr-esfahani, Christophe Klein

**Affiliations:** 1 Acquired Immunodeficiency Research Center, Isfahan University of Medical Science, Isfahan, Iran; 2 Center for Chronic Immunodeficiency, University Hospital, Freiburg, Germany; 3 Isfahan Fertility and Infertility Center, Isfahan, Iran; 4 Department of Genetics and Molecular Biology, School of Medicine, Isfahan University of Medical Science, Isfahan, Iran; 5 Mashhad University of Medical Sciences, Mashhad, Iran; 6 Department of Reproductive Biotechnology, Reproductive Biomedicine Research Center, Royan Institute for Biotechnology, ACECR, Isfahan, Iran; 7 Department of Pediatric, Children’s Hospital, University Hospital, LMU, Munich, Germany

**Keywords:** Ataxia telangiectasia, CGD, CVID, Conception, Fertility, PGD, Pregnancy, Primary immunodeficiency disease, Wiskott-Aldrich

## Abstract

**Background:**

Patients with primary immunodeficiency disease (PID) who survive to adulthood and willing to have a child mostly are worried whether their disease affects their fertility and/or pregnancy and also if their child would be predisposed to PID.

**Case presentation:**

We report the outcome of conception, pregnancy and their management in 9 families with definite diagnosis of PID. A chronic granulomatous disease subject with an uneventful pregnancy developed fungal sacral osteomyelitis few weeks after delivery. A pregnant common variable immunodeficiency disease (CVID) patient with idiopathic thrombocytopenia had platelet count dropped before delivery. A sever neutropenic mother who refused to get IFNγ delivered two healthy children. A CVID case intolerant to IVIg with eclampsia and PTE delivered a baby. Another CVID female gave birth to a baby without being on any treatment since she was not diagnosed with immunodeficiency disease at that time. A healthy girl was implanted via preimplantation gender selection in a family who owned a Wiskott Aldrich-affected son. A family who had two children with Ataxia Telangiectasia used donated oocyte for their 3rd child. Prenatal genetic diagnosis was used to screen the fetus for the impaired BTK and CVID genes detected in sibling and father respectively in 2 separate families.

**Conclusion:**

Pregnancy in PID patients is more complex than normal population. Because, not only it has the chance of being inherited by the offspring, but also there are some risks for the mother if she has any kind of immunity component defects. So consultation with a clinical geneticist is crucial to choose the best available approach. They also should be observed and followed by a clinical immunologist to take the best possible safe care.

## Background

Primary immunodeficiency diseases (PID) are a group of inherited disorders in which components of the immune system are missing or do not function properly [1]. Patients usually experience their first symptoms during childhood, although some types may not be recognized until later in life. Individualized treatment of the burden of PID has reduced the mortality and morbidity, so patients often survive into adulthood. The wish to conceive in PID affected parents may be negatively influenced by the fear of complications and concerns about inheritance patterns. Some cases with common variable immunodeficiency disease (CVID) have been studied during pregnancy period but it has been much more focused on the treatment rather than prevention of giving birth to a PID child and/or complications due to pregnancy. Pregnancy in other PID types except CVID is not studied much. This study presents some families with definite diagnosis of PID who conceived naturally or by in-vitro fertilization (IVF) with the consultation of a clinical geneticist, they were tightly observed and managed by the obstetrician and the clinical immunologist during their pregnancy and also were followed for any likely complication due to pregnancy.

## Case presentation

### Case 1

A 23 year-old patient with Chronic Granulomatous Disease (CGD) gave birth to a normal healthy male. During pregnancy IFNγ was discontinued and through control visits acute phase reactants were evaluated with no increase. Diagnosis of CGD was established at 8 years old due to recurrent pneumonia, empyema, oral ulcers, skin abscess and cervical mycobacterial lymphadenitis. Genetic analysis revealed p47-phox gene mutation. She was born out of consanguineous marriage and her sister had been diagnosed with CGD as well. Four weeks after delivery she went to emergency department complaining of low back pain irradiated to left gluteal, sacral and lumbosacral area. Laboratory workup showed leukocytosis (WBC = 11,700 cell/mcL) and increased levels of liver enzymes (SGPT = 131 U/L, normal:7–55, SGOT = 56 U/L normal:8–45, ALP = 1588 U/L, normal:45–115). Abdominopelvic US revealed granulomatous lesions in uterus and liver. Contrast lumbosacral MRI showed enhancement in L5 and S1 suggesting infection or inflammation. Pathology Department reported granulomatous lesion and PCR positive for *Aspergillus fumigatus*. She was diagnosed with fungal osteomyelitis and went under treatment with IFNγ and Voriconazole for 12 weeks with good response. In follow-up tests liver enzymes decreased (SGPT = 54 U/L, SGOT = 24 U/L, ALP = 423 U/L) and granulomatous lesions disappeared.

### Case 2

**Fig. 1:**
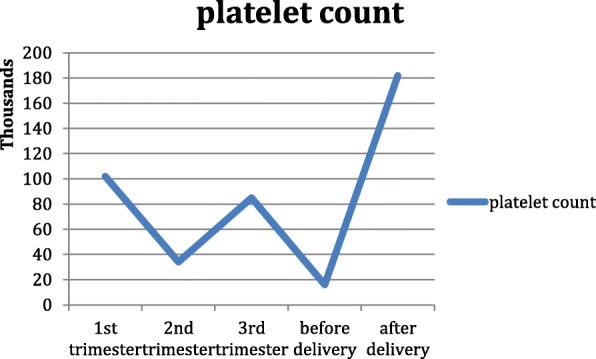
Platelet count in different gestational age

A 23 year-old patient with CVID whose diagnosis was done 7 years before pregnancy when she was 16 years old with clinical history of lower and upper recurrent respiratory tract infections and autoimmune thrombocytopenia. Genome analysis showed heterozygous missense variant in exon 3 of CDX1 gene. Blood tests at 5 weeks of gestation showed IgM of 0.8 g/L (0.3–2.5), a total IgG of 5.6 g/L (normal 6.0–16.0), and IgA of 0.7 g/L (normal 0.8–5). During pregnancy she received 500 mg/kg of IVIg every 3 weeks. In the 2nd trimester of pregnancy she presented idiopathic thrombocytopenia, which was managed with higher doses of IVIg (800 mg/kg) with no clinical manifestation. Platelet count dropped to 16x10^3^platelets/mm^3^ abruptly before her cesarean section and need hindered urgent platelet infusion before delivery. Ultimately she gave birth to a full term, normal healthy child and few days after delivery platelet count reached normal level (Fig. 1).

### Case 3

**Fig. 2:**
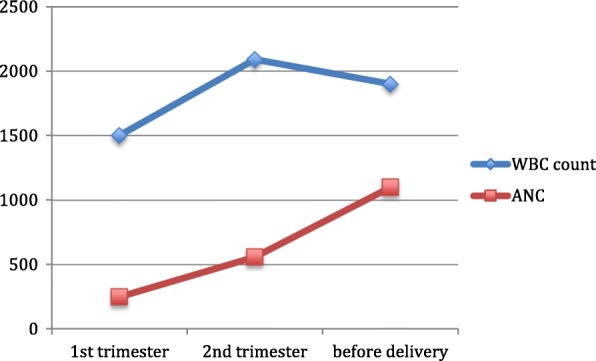
WBC count and ANC in different gestational age

A patient with JAGN1 gene mutation suffering from Severe Congenital Neutropenia (SCN). Initially she was evaluated for recurrent pneumonia, visceral abscesses, oral ulcers and severe dental problems at 23 years old. Her first pregnancy was at 25-year-old with no complication. She gave birth to a healthy male whose genomic analysis showed he is not a carrier. She became pregnant again at the age of 37 and just delivered a healthy baby. The patient refused to receive G-CSF all these years and has attended the clinic with pneumonia, hemoptysis and a drop in her ANC twice in her 2nd pregnancy at 2nd and 3rd trimester accompanied with severe neutropenia (Fig. 2). Each time she received antibiotics for pneumonia and became well again. At 38w of gestation she fell down from stairs and her fibula has fractured. Prophylactic antibiotics were administered in order to avoid progression to osteomyelitis. Her neutrophil count was in normal range during hospitalization and delivery (ANC > 1500). She breast-fed her baby from birth and the baby is infection-free and thriving, with normal neutrophil count.

### Case 4

A 29 year-old woman, a case of CVID who was unrecognized till age 22. She had autoimmune hepatitis (AST=) with false negative markers due to immunodeficiency. Biopsy from duodenum showed celiac-like disease. She was on immunosuppressant drugs before her definite diagnosis. Her genetic result revealed NF-κB2 frameshift mutation. She had history of recurrent cold, hospitalization for pneumocystis jiroveci, atopy and allergy. Immunoglobulin levels were as follows; IgG = 7.05 g/L (normal: 6–16), IgM = 0.3 g/L (normal: 0.5–2.5), IgA = 0.5 g/L (normal: 0.8–4), IgE = 2 g/L (normal < 0.002). Flowcytometry result demonstrated CD4 = 59%, CD8 = 14%, CD16 + 56 = 14%. While she was started on IVIg therapy she showed allergic reaction to the product. IVIg was discontinued and the patient was reluctant to take it anymore. She was returned to use immunosuppressive drugs including cellcept and prednisone. She became pregnant and all drugs were discontinued. Strangely her liver enzymes became normal (AST = 20, ALT = 32). Eclampsia developed at 24w of gestation, handled under supervision of her obstetrician. She received heparin for 1 month as she had tachypnea and dyspnea and was suspicious to PTE at 30w. Her Immunoglobulin level at 34w of gestation was IgG = 5.1 g/L and IgM = 0.42 g/L and IgA = 0.49 g/L. She has born her child at 38w. The baby is well and we are waiting for her immunoglobulin level and other laboratory results to decide on vaccination.

### Case 5

A 26 year-old woman is recently known for CVID. She was referred to our clinic for extensive bilateral bronchiectasis. Her laboratory results showed hypogammaglobulinemia (IgM = 0.0.14 g/L normal: 0.4–2.6, IgG = 4.1 g/L normal: 6.5–18, IgA = 0.05 g/L normal: 0.7–3.6). She is getting IVIg now but she became pregnant 2 years ago while her diagnosis was not made and no immunotherapy was given. She had severe dyspnea and suffered from recurrent pneumonia during her pregnancy that was treated with antibiotic. She gave birth to a healthy boy baby. We do not have the immunoglobulin level of the child at birth but according to the mother’s statement he had no serious infection in his first 6 months of life.

### Case 6

Male patient with CVID born out of a consanguineous marriage. Whole exome sequencing revealed 2 heterozygous mutations in TNFRSF13B gene. This male patients married a healthy appeared woman and his wife became pregnant. During pregnancy, his wife underwent chorionic villus sampling (CVS), fetal DNA was obtained and revealed homozygous mutation in TNFRSF13B gene suggesting a strange fact that the mother is carrier of a mutation in the same gene while she is not a family member of the father. The boy infant was born and advised not to receive any live attenuated vaccine. He was brought to our clinic for further evaluation and hypogammaglobulinemia was found in his lab data in his 6-month-old visit (IgG = 1.5 g/L, IgA = 0.1 g/L, IgM = 0.2 g/L).

### Case 7

A male child with meningitis, recurrent upper and lower respiratory infections was diagnosed with X-linked Agammaglobulinemia known as Bruton’s tyrosine kinase (Btk) deficiency. The parents conceived their second child before genetic counseling. Chorionic villus sampling (CVS) was performed in 12 weeks gestation, DNA is extracted and sequenced. The mutation exists in the fetus as well. Fetal DNA sample was checked for maternal DNA contamination by short tandem repeats markers. Although in this case abortion was legally allowed, the parents chose to preserve the boy offspring. We only advised them avoid live vaccination for him.

### Case 8

Two siblings were born from a consanguineous union and diagnosed with Ataxia-Telangiectasia during infancy. The boy died from gastric cancer in early childhood but the girl overcame recurrent upper and lower respiratory infections by monthly IVIg. After genetic counseling parents decided to have another baby using a donor egg. The recipient couple underwent standard IVF process. Collected oocytes were inseminated via ICSI (intracytoplasmic sperm injection). Among them 3 fertilized successfully 4 days later and one embryo was transferred to the prepared uterine of the recipient. CVS was performed at 12w of gestation and genetic result was normal.

### Case 9

Patient diagnosed with Wiskott-Aldrich syndrome due to thrombocytopenia, anemia, severe eczema, molluscum contagiosum and hypogammaglobulinemia. Genetic analysis revealed homozygous mutation in exon 8. The parents received genetic counseling about inheritance pattern of the disease and they chose to have another baby via PGD gender selection. In this process, hormonal stimulation followed by oocyte aspiration is required for IVF. Blastomere from an 8-cell human cleavage-stage embryo was biopsied on day 4 after IVF and single cell PCR was applied to provide information about the sex chromosomes of the embryo. An XX embryo was transferred to uterine cavity and now they own a 2-month-old healthy daughter.

## Discussion

Most PIDs are inherited in one out of the three patterns of inheritance including autosomal recessive (AR), X-linked recessive (XR) and autosomal dominant (AD). In the XR inheritance pattern which asymptomatic parents have an affected son it has been suggested to select the child gender prior to conception. According to Islamic law, sex selection for X-linked disorders is allowed [2, 3], as we have done for case 8 which was affected by WAS.

Regarding the concerns of the parents who have the disease or have an affected child, some methods to determine the chance of inherit the disease to the child before delivery are available**.** Preimplantation genetic diagnosis (PGD) is one of the useful established procedures for couples who are at high risk of bearing a child with single gene disorder. Implantation of an unaffected embryo via IVF in individuals with SCID, LAD, XL-agammaglobulinemia and AT have been previously reported [4–7]. Evidences supporting the efficacy of PGD in detection of some immunodeficiencies such as Wiskott-Aldrich syndrome, X-linked hyper-IgM syndrome (HIGM), X-linked hypohidrotic ectodermal dysplasia with immune deficiency (HED-ID), ataxia telangiectasia and Omenn syndrome and transferring healthy embryos are also available [8]. Gamete donation suggestible for infertile couples could assist affected volunteer or carrier parents to ascertain wellness of their baby [9]. Couples at risk could benefit from prenatal diagnosis via chorionic villus sampling (CVS) and amniocentesis (12-16w) early in pregnancy. Prenatal genetic analysis in fetuses born to PID carriers allows them to choose the optimal management before childbirth [10]. Additionally CVS had been applied to ascertain the gender of the fetus in X-linked immunodeficiencies such as WAS, X-CGD, IPEX, XLA and SCID [10–12]. Early diagnosis of life-threatening congenital disease provides the chance of stop pregnancy. A couple assessed for CD40L deficiency (HIGM) opted for voluntary abortion to avoid giving birth to an affected child [13].

It is important to know that patients with severe combined immunodeficiency, hyper IgM and Wiskott-Aldrich should be kept away from all live vaccines such as BCG and OPV. Patients with CVID are also not recommended to be vaccinated with OPV and VZV. Newborns with suspected immunodeficiency who did not have definite prenatal diagnosis or have an older sibling with one of the PIDs mentioned above should avoid use of live vaccines [14].

This patient was affected by sacral bone osteomyelitis. Among PIDs, CGD holders show the highest susceptibility for *Aspergillus* osteomyelitis with the most frequent sites of infection being vertebrae, cranium, ribs and long bones [15–17]. It has been suggested that infections with *Aspergillus nidulans* in CGD cases could be acquired by hematogenous dissemination [16]. Osteomyelitis of pubis symphysis in pregnancy has been previously reported [18, 19], sporadic cases of osteomyelitis in vertebra and iliac bone with gram-positive cocci, MRSA and Salmonella, as well [20–22]. It has been hypothesized that pregnancy sacroiliitis could be associated with microscopic areas of injury on the joint surfaces produced by the changes during pregnancy [23]. Antibacterial and antifungal prophylaxis is the cornerstone of prevention in CGD patients. IFNγ is an immunotherapy administered in patients with CGD but it is assigned to pregnancy category C by the FDA. Studies on primates have shown that it causes abortion at 100 times the human dose. As there is no controlled data in human pregnancy, it has been held in our case while she became pregnant. Also there is no evidence on the excretion of interferon gamma-1b into human milk and we avoid using the drug in breastfeeding. This patient developed granuloma in visceral organs after cessation of IFNγ and healed after re-administration of the drug. In recent years, the routine use of antimicrobial prophylaxis such as trimethoprim-sulfamethoxazole (TMP-SMX) and newer azole drugs such as posaconazole and isovuconazole has reduced the mortality and morbidity of patients with CGD but a major concern of a pregnant woman is teratogenicity of the drugs. Although Amphotericin B has known toxicity but still is the first-choice parenteral drug for fungal infection during pregnancy. Topical drugs can be safely applied due its limited absorption, but most oral azoles are found to have teratogenic effects throughout the first trimester especially high-dose fluconazole. Although recent analysis revealed additional safety data on using itraconazole and lipid derivatives of Amphotericin B, there is still no proven antifungal prophylactic agent known to be completely safe in pregnancy [24–26]. We can take benefit of Cotrimoxazole in second trimester and even in early 3rd trimester, but TMP-SMX is contraindicated in first and third trimester of pregnancy and it would be better to use penicillin or 3rd generation of cephalosporin as antibacterial prophylactic treatment in this period [25].

Regarding to predominantly antibody deficiencies an approved protocol on the management of CVID or HIGM in pregnancy does not exist, there are some case reports of success [27]. It is recommended to use exogenous intravenous IgG (IVIg) in CVID mothers during pregnancy and breastfeeding [28–31]. It has been demonstrated that Regular IVIG therapy (RIT) should be administered in early stages of pregnancy since it takes 3–6 months to achieve the protective level of IVIg in the mother and the infant [26, 32]. In patients with decreased level of immunoglobulins, without RIT transfer of IgG to the fetus may decrease. Lack of sufficient protective antibody predisposes fetus to intrauterine infections. Also the risk of infection in early childhood is increased as humoral immune system of newborn is not mature enough. Due to haemodilution during pregnancy there is a depletion in the level of maternal IgG concentration. So, higher dose of IVIG is required to reach the placenta. It is also suggested to do a booster dose of IVIG prior to delivery regarding the patients’ IgG trough levels. Routinely 0.4–0.6 g/kg is administered in pregnant women [30, 31]. There was a study reported 2 pregnant CVID women who declined to receive IVIg, although IgG levels of mothers were low, fetuses maintain normal IgG level. The theory behind the fact was increasing the transport of IgG from mother to fetus, when maternal IgG is low by upregulation of IgG Fc receptors in the endosome of the syncytiotrophoblast [33].

## Conclusion

For PID patients, pregnancy is more complicated because not only the child can inherit the disease but also the mothers are at risk because of defects in the immune systems. So genetic counseling is mandatory. This will allow PID affected families to find the best available approach. Management of the disease during and after pregnancy should be monitored by a clinical immunologist to ensure the best safe care.

## Abbreviations

ANC: Absolute Neutrophil Count
BTK: Bruton Tyrosine Kinase
CGD: Chronic Granulomatous Disease
CVID: Common Variable Immunodeficiency Disease
IPEX: Immunodysregulation Polyendocrinopathy Enteropathy X-linked)
LAD: Leukocyte Adhesion Deficiency
PID: Primary Immunodeficiency Disorder
WAS: Wiskott- Aldrich Syndrome

## References

[1] Bonilla FA, Geha RS (2003). 12. Primary immunodeficiency diseases. J Allergy Clin Immunol.

[2] Dezhkam L, Dezhkam H, Dezhkam I. Sex selection from Islamic point of view. Iran J Reprod Med. 2014;12(4):289-90. PubMed PMID: 24976826 PMC4071636 24976826

[3] Eftekhaari TE, Nejatizadeh AA, Rajaei M, Soleimanian S, Fallahi S, Ghaffarzadegan R (2015). Ethical considerations in sex selection. J Educ Health Promot.

[4] Hellani A, Lauge A, Ozand P, Jaroudi K, Coskun S (2002). Pregnancy after preimplantation genetic diagnosis for Ataxia telangiectasia. Mol Hum Reprod.

[5] Lorusso F, Kong D, Jalil AK, Sylvestre C, Tan SL, Ao A (2006). Preimplantation genetic diagnosis of leukocyte adhesion deficiency type I. Fertil Steril.

[6] Tomashov-Matar R, Biran G, Lagovsky I, Kotler N, Stein A, Fisch B (2012). Severe combined immunodeficiency (SCID): from the detection of a new mutation to preimplantation genetic diagnosis. J Assist Reprod Genet.

[7] Xu C, Xu B, Huang H, Huang X, Jin F (2009). Preimplantation genetic diagnosis for X-linked agammaglobulinemia: a case report. Fertil Steril.

[8] Verlinsky Y, Rechitsky S, Sharapova T, Laziuk K, Barsky I, Verlinsky O (2007). Preimplantation diagnosis for immunodeficiencies. Reprod BioMed Online.

[9] Klein J, Sauer MV (2002). Oocyte donation. Best Pract Res Clin Obstet Gynaecol.

[10] Lee WI, Huang JL, Yeh KW, Cheng PJ, Jaing TH, Lin SJ (2016). The effects of prenatal genetic analysis on fetuses born to carrier mothers with primary immunodeficiency diseases. Ann Med.

[11] Bai QL, Liu N, Kong XD, Xu XJ, Zhao ZH (2015). Mutation analyses and prenatal diagnosis in families of X-linked severe combined immunodeficiency caused by IL2Rgamma gene novel mutation. Genet Mol Res.

[12] Siminovitch KA (2003). Prenatal diagnosis and genetic analysis of Wiskott-Aldrich syndrome. Prenat Diagn.

[13] Torok O, Toth B, Erdos M, Csorba G, Gyimesi E, Balogh I (2012). Molecular diagnostic challenges and complex Management of Consecutive Twin Pregnancies in a family with CD40 ligand deficiency. Scand J Immunol.

[14] Sobh A, Bonilla FA (2016). Vaccination in primary immunodeficiency disorders. J Allergy Clin Immunol Pract.

[15] Dotis JRE (2004). Osteomyelitis due to aspergillus spp. in patients with chronic granulomatous disease: comparison of aspergillus nidulans and aspergillus fumigatus. Int J Infect Dis.

[16] Gamaletsou MN, Rammaert B, Bueno MA, Moriyama B, Sipsas NV, Kontoyiannis DP (2014). Aspergillus osteomyelitis: epidemiology, clinical manifestations, management, and outcome. J Infect.

[17] Margolis DM, Melnick DA, Alling DW, Gallin JI (1990). Trimethoprim-sulfamethoxazole prophylaxis in the management of chronic granulomatous disease. J Infect Dis.

[18] Gamble K, Dardarian TS, Finstein J, Fox E, Sehdev H, Randall TC (2006). Osteomyelitis of the pubic symphysis in pregnancy. Obstet Gynecol.

[19] Knoeller SM, Uhl M, Herget GW (2006). Osteitis or osteomyelitis of the pubis? A diagnostic and therapeutic challenge: report of 9 cases and review of the literature. Acta Orthop Belg.

[20] Anderson BL, Nau GJ, Simhan HN (2007). Idiopathic vertebral abscess in pregnancy: case report and literature review. Am J Perinatol.

[21] Nguyen LN, Lopes C, Folk JJ (2015). Vertebral osteomyelitis in pregnancy from a methicillin-resistant staphylococcus aureus vulvar abscess. a case report. J Reprod Med.

[22] Agustsson AI, Olafsson K, Thorisdottir AS (2009). Salmonella osteomyelitis in pregnancy. Acta Obstet Gynecol Scand.

[23] Haq I, Morris V (2001). Post-partum septic sacroiliitis. Rheumatology.

[24] Pilmis B, Jullien V, Sobel J, Lecuit M, Lortholary O, Charlier C (2015). Antifungal drugs during pregnancy: an updated review. J Antimicrob Chemother.

[25] Leiding JW, Holland SM, Adam MP, Ardinger HH, Pagon RA, Wallace SE, LJH B, Mefford HC (1993). Chronic Granulomatous Disease.

[26] Schaffer FM, Newton JA (1994). Intravenous gamma globulin administration to common variable immunodeficient women during pregnancy: case report and review of the literature. J Perinatol: official journal of the California Perinatal Association.

[27] Osada H, Morikawa Y, Nishiwaki T, Sekiya S (1996). Intravenous immunoglobulin replacement therapy for common variable immunodeficiency during pregnancy. Arch Gynecol Obstet.

[28] Palmeira P, Costa-Carvalho BT, Arslanian C, Pontes GN, Nagao AT, Carneiro-Sampaio MM (2009). Transfer of antibodies across the placenta and in breast milk from mothers on intravenous immunoglobulin. Pediatr Allergy Immunol.

[29] Gardulf A, Andersson E, Lindqvist M, Hansen S, Gustafson R (2001). Rapid subcutaneous IgG replacement therapy at home for pregnant immunodeficient women. J Clin Immunol.

[30] Vitoratos N, Bakas P, Kalampani H, Creatsas G (1999). Maternal common variable immunodeficiency and pregnancy. J Obstet Gynaecol.

[31] Shalev E, Ben-Ami M, Peleg D (1993). Common variable hypogammaglobulinemia in pregnancy. Br J Obstet Gynaecol.

[32] Manson AL, Zaheri S, Kelleher P, Wakelin S, Nelson-Piercy C, Seneviratne SL (2012). Management of granulomatous common variable immunodeficiency diagnosed in pregnancy: a case report. J Perinatol: official journal of the California Perinatal Association.

[33] Nagendran V, Emmanuel N, Bansal AS (2015). Does the maternal serum IgG level during pregnancy in primary antibody deficiency influence the IgG level in the newborn?. Case Rep Immunol.

